# Barriers and facilitators to the access to and use of formal dementia care: findings of a focus group study with people with dementia, informal carers and health and social care professionals in eight European countries

**DOI:** 10.1186/s12877-018-0816-1

**Published:** 2018-06-04

**Authors:** Astrid Stephan, Anja Bieber, Louise Hopper, Rachael Joyce, Kate Irving, Orazio Zanetti, Elisa Portolani, Liselot Kerpershoek, Frans Verhey, Marjolein de Vugt, Claire Wolfs, Siren Eriksen, Janne Røsvik, Maria J. Marques, Manuel Gonçalves-Pereira, Britt-Marie Sjölund, Hannah Jelley, Bob Woods, Gabriele Meyer, on behalf of the Actifcare Consortium, Frans Verhey, Marjolein de Vugt, Claire Wolfs, Ron Handels, Liselot Kerpershoek, Gabriele Meyer, Astrid Stephan, Anja Bieber, Anja Broda, Gabriele Bartoszek, Bob T. Woods, Hannah Jelley, Martin Orrell, Anders Wimo, Anders Sköldunger, Britt-Marie Sjölund, Knut Engedal, Geir Selbæk, Mona Michelet, Janne Røsvik, Siren Eriksen, Kate Irving, Louise Hopper, Rachael Joyce, Orazio Zanetti, Elisa Portolani, Manuel Gonçalves-Pereira, Maria J. Marques, M. Conceição Balsinha, Helena Bárrios, Ana Machado

**Affiliations:** 1 Institute for Health and Nursing Science, Martin Luther University Halle-Wittenberg, Magdeburger Straße 8, 06112 Halle (Saale), Germany; 2 School of Nursing and Human Sciences, Dublin City University, Glasnevin, Dublin 9, Ireland; 3 Alzheimer Unit, IRCCS S. Centro Giovanni di Dio “Fatebenefratelli”, Via Pilastroni 4, Brescia (BS), Italy; 4 Alzheimer Center Limburg, Maastricht University, Maastricht, The Netherlands; 5 Norwegian National Advisory Unit on Ageing and Health, Vestfold Hospital Trust, Tønsberg, Norway; 6 Department of Geriatric Medicine, Oslo University Hospital, Aldring og Helse, Oslo, Norway; 7 Chronic Diseases Research Center, Nova Medical School | Faculdade de Ciências Médicas, Universidade Nova de Lisboa, Campo Mártires da Pátria, 130, 1169-056 Lisbon, Portugal; 8 Faculty of Health and Occupational Studies, Department of Health and Caring Sciences, University of Gävle, Gävle, Sweden; 9 Dementia Services Development Centre Wales, Bangor University, Bangor, LL57 2PZ UK

**Keywords:** Dementia, Person with dementia, Informal carer, Formal care, Utilisation, Focus groups

## Abstract

**Background:**

People with dementia and informal carers often access formal care late in the process of dementia. The barriers and facilitators to service use from the perspectives of different stakeholders involved are not well understood. Thus, we aimed to explore the barriers and facilitators of access to and utilisation of formal care from the perspectives of people with dementia, their informal carers and health and social care professionals.

**Method:**

Focus groups with people with dementia, informal carers and professionals were conducted in eight European countries. Recruitment targeted people with dementia, informal carers with experience of formal care and professionals involved in providing (access to) formal care. Qualitative content analysis using open coding was used on a national level. Cross-national synthesis was based on the translated national reports.

**Results:**

Overall, 55 focus groups with 261 participants were conducted, involving 51 people with dementia, 96 informal carers and 114 professionals. Sixteen categories describing barriers and facilitators were identified, referring to three global themes: Aspects related to 1) individuals involved, 2) the system or 3) overarching aspects. The attitudes and beliefs of people with dementia and their carers may have a major impact, and they often serve as barriers. Formal care was perceived as a threat to the individual independence of people with dementia and was thus avoided as long as possible. A healthcare professional serving as a constant key contact person could be an essential facilitator to overcome these barriers. Contact should be initiated proactively, as early as possible, and a trusting and consistent relationship needs to be established. Beyond that, the findings largely confirm former research and show that barriers to accessing and using formal care still exist across Europe despite a number of national and European initiatives.

**Conclusion:**

Further investigations are needed to elaborate how the concept of a key contact person could be integrated with existing case management approaches and how the independence and autonomy of people with dementia can be strengthened when formal care needs to be accessed and used. These may be meaningful facilitators regarding enhanced access to formal care for people with dementia and their families.

**Electronic supplementary material:**

The online version of this article (10.1186/s12877-018-0816-1) contains supplementary material, which is available to authorized users.

## Background

Compared with other chronic conditions, dementia is more often a reason for care dependency among older people [1]. People with dementia may become dependent on help in several everyday activities early in the progression of the disorder [2] and can have a wide range of needs, as too can their informal carers [3]. Several types of support and community services may become necessary as the condition progresses [1, 4]. In contrast, research has shown that people with dementia and their informal carers use fewer services in comparison to other people in need of care [5, 6]. The findings of one systematic review show that medical services are frequently used, whereas community services, such as home support, day care, respite care or counselling, are used less often, even though these services may be particularly helpful [7].

The point in time when people with dementia and their informal carers gain access to professional support and care may have implications for how the caregiving situation develops. Research suggests that the first phases of the caregiving process are particularly important and that the timely use of community services potentially delays institutionalisation [8].

The results of an early study indicated that the most important reason for the non-use of services was that informal carers did not consider services as necessary, although a considerable level of burden was reported. The reluctance of the person with dementia and a lack of knowledge about the services also contributed to non-utilisation [9, 10].

Since then, the number of studies investigating the use of services by people with dementia has increased. Stigmatic beliefs about dementia and inadequate knowledge were found to be main barriers preventing people from seeking help [11]. Recent systematic reviews focus on factors influencing the service use of people with dementia and their informal carers, including community-dwelling people with dementia in general [7, 12], only certain types of services such as respite care [6, 13], or they exclusively investigate male carers [14]. The majority of these studies were observational studies or surveys conducted in the USA, Canada and Australia, seeking to explain factors influencing the use of services by comparing people who are using services with those who do not. Moreover, the findings regarding diverse types of services are often contradictory [12, 13].

Recent qualitative studies add to the scope of knowledge and explore the process of seeking help and using services but from the perspectives of informal carers only [15, 16]. These studies show that informal carers may sometimes perceive accessing and navigating health- and social care services as even more burdensome than caregiving itself. Successful interaction between formal and informal care is shaped by the trusting relationship and recognition between professionals and informal carers [17], and it requires improved professional understanding of the complex social relationships and functioning of families [18].

A scoping review that we performed of studies investigating access to and use of services for people with dementia and their families shows that people with dementia are rarely included in studies investigating service use, and if so, they often only serve as informants regarding cognitive or functional and behavioural status. Very few studies have sought to understand barriers in service use from the perspectives of people with dementia themselves, i.e., a case study focusing on certain ethnic groups or early onset dementia or that investigates service needs (unpublished work, manuscript submitted).

A qualitative study including the perspectives of all stakeholders involved is lacking but may be particularly important in order to comprehensively describe perceived barriers and facilitators in accessing and using formal community care for people with dementia. Therefore, we explored the perspectives of all stakeholders involved in the process of accessing and using formal care and services, namely, people with dementia, their informal carers and health and social care professionals. We aimed to improve the understanding of the facilitators and barriers to the access to and the use of formal dementia care for the further development of appropriate services and interventions.

## Methods

The transnational research project Actifcare (ACess to Timely Formal Care, http://www.actifcare.eu/) investigated the accessibility and utilisation of formal care for people with dementia and their informal carers in eight European countries: Germany (DE), Ireland (IE), Italy (IT), the Netherlands (NL), Norway (NO), Portugal (PT), Sweden (SE) and the United Kingdom (UK). The main aim of the study is to analyse the pathways to care and to gain a better understanding of the reasons for inequalities in access to health and social care. Thus, a cohort study with dyads of people with dementia and their informal carers over a one-year period was performed, and their needs, service use patterns and quality of life were assessed [19]. To further explore the barriers and facilitators to accessing and using formal care—as experienced by people with dementia and their carers and health and social care professionals—a cross-national focus group study was conducted (Work Package 2 of the Actifcare Project). Focus groups are considered an appropriate approach, as they have the potential to enhance the understanding of factors that influence behaviour or motivations [20, 21]. The findings of the focus groups have also been used to inform subsequent parts of the Actifcare study, in particular, single interviews with national political decision makers/influencers and semi-structured interviews with a subsample of the dyads from the cohort study.

Formal dementia care was defined in accordance with the overall research protocol. It comprises home nursing care; day care services; and community, long-term medical and social care structures, such as respite services, multi-professional mental health teams and dementia care teams. It does not include acute medical services such as hospitals or specialised physicians. It also excludes domestic home help, housekeepers, volunteers, support groups, transport services and meal programs [19].

### Group composition and participants

Separate focus groups were performed with people with dementia, informal carers and healthcare professionals. Focus groups typically consist of five to eight participants [20]. For the focus groups with older and cognitively impaired people, we decided that smaller groups would be more appropriate, so three to four participants were included. According to the study protocol of the Actifcare project, all types of dementia were included. People with dementia had to be formally diagnosed. The phase of dementia was not assessed prior to the focus groups. Informal carers took care of people with dementia from early to advanced stages of the disease.

We included people with dementia and informal carers who had some kind of experience with accessing and using formal care or who had at least tried to access formal care in the past.

Informal carers could be relatives, friends or neighbours. The intention was for the sample to cover a wide range of caregiving experiences, along with different ages and different relationships to the person with dementia. Informal carers had to have been in regular contact with the people with dementia and involved in care decision making. Former informal carers were included, since they could report a wide range of caregiving experiences.

Healthcare professionals had to be in regular contact with people with dementia and/or informal carers during their daily work, either providing access to formal care or providing formal care and support for people with dementia and their families. Relevant professional backgrounds were determined by each country based on the inter-disciplinary nature of dementia care and access to dementia care in that country, which was investigated in a preceding phase of the Actifcare project [22].

### Sampling

Participants were contacted by gatekeepers (such as counselling agencies, support groups and known contact persons from other parts of the project) and selected according to the criteria described above, with the aim of having a large variation in perspectives. Oral and written information about the study was provided by the researchers.

### Data collection

An interview guide was developed in close collaboration with all partners to ensure the applicability across the countries, and it was piloted in Germany (09/2014). The interview guide was developed following a comprehensive understanding of access to care [23] and thus focused on expectations or concerns prior to using formal care and personal experiences with accessing formal care, as well as barriers and facilitators to the access to and utilisation of formal care. The topic guide was slightly different per type of individual, but it followed the same global topics (Additional file 1). In line with the pilot study, it was found that discussing issues regarding formal care with people with dementia was challenging. To ease the discussion and clarify the topic, pictures showing typical caregiving situations were added to the questions for people with dementia [24].

The questions were carefully translated by each country’s research team into their national language. Two experienced and trained researchers conducted the focus groups in each country; one served as a moderator who fostered an active and open discussion, and the other served as an assistant who took notes (field work took place between 09/2014 and 04/2015). All focus groups were audio recorded and transcribed intelligent verbatim (i.e., omitting filler expressions, redundant phrases or words) for the purpose of content analysis.

### Data analysis

So far, established guidelines on how to analyse cross-national qualitative data are lacking. Some published cross-national studies have applied predetermined categories [25, 26]. We argue that predetermined categories may not appropriately reflect the diversity across the countries; therefore, qualitative content analysis using open coding was performed in each county [27, 28]. National findings were translated into English and synthesised consecutively by the Work Package leading team (DE). Overall, we followed an interpretive paradigm [29].

#### National analysis

To ensure consistency and methodological rigor [25], a manual was provided to all the partners (*available on request from the corresponding authors*). To extract the content, meaning units (e.g., sentences, paragraphs) were labelled with codes. Based on the codes, categories and sub-categories were developed that were considered the manifest content of the transcripts [28]. The categories for the three types of focus groups were derived separately. We followed the assumption that during this process, dialogue among researchers promotes the most likely interpretation of the data [28]. Thus, two researchers per country independently derived initial codes based on the most information-rich transcripts. Categories were then developed during a personal meeting. The remaining material was then analysed by one researcher applying the categories. In a final step, the categories were introduced to researchers not involved in the analysis in order to check the plausibility. The national findings (in terms of categories, descriptions and anchor examples) were translated into English. To increase the trustworthiness of the translations, we followed the manual recommendations stipulating that the same person should perform all the translational work in each country [30].

#### Cross-national analysis

The translated national findings were synthesised using a comparable approach as the one used in the national analysis. Two researchers of the Work Package leading team (DE) independently derived first codes based on two country reports, and first cross-national categories were developed during a joint meeting. The remaining reports were then analysed by using the derived categories; modifications were possible throughout the analysis. As a final step, the system of categories was reviewed and the categories were sorted into global themes. To approve the synthesis and to guarantee that no country-specific findings were misinterpreted, the categories were discussed by all the partners using written feedback, video conferences and personal project meetings that took place twice per year. The analysis was supported by the software MAXQDAplus version 11 (VERBI GmbH, Berlin, Germany).

## Results

### Description of the focus groups

Overall, 55 focus groups were performed. Each lasted, on average, approximately 90 min (range 26–140 min); those with people with dementia tended to be shorter and lasted approximately 70 min (range 26–120 min). The mean group size was five participants (range 2–8 participants; only one focus group with people with dementia consisted of two participants).

### Participants

In total, 261 participants took part in the focus groups (Table 1). People with dementia and their informal carers used diverse services, including support groups, counselling, educational training, help at home with social activities or personal care, community day centres, day care, memory clinic, and respite care. In Ireland, the United Kingdom, the Netherlands and Norway, interdisciplinary teams were in place in the community that took care of a number of participants. Only three informal carers and one person with dementia did not use any type of service. Four people with dementia in Portugal lived in a nursing home and reported their experiences retrospectively. In Portugal, only a few community services are available, and institutional care is often the first type of service. In most countries, it was difficult to identify eligible people with dementia who were able and willing to join a focus group. Since younger people with dementia and people in an early stage of dementia were easier to engage with, they were predominately included in some countries. Informal carers represented different caregiving situations and different relationships to the people with dementia and were more often female. Health and social care professionals had a mean working experience of 16 years and were mainly nurses, although there were also psychologists, social workers, general practitioners (GPs) and other physicians.

**Table 1:** Overview of focus groups and characteristics of participants per country. Values are numbers unless stated otherwise

People with dementia	Total	DE	IE	IT	NL	NO	PT^c^	SE	UK
Number of focus groups	14	3	2	2	2	3	1		1
Number of participants	51	10	7	6	8	9	4		7
Number of female participants	28	7	6	4	2	4	3		2
Mean age, years (range)	76 (54–96)	68 (55–84)	68.5 (54–88)	80 (75–87)	76 (64–85)	75 (61–86)	90 (82–96)		75 (66–85)
Mean time since diagnosis, years (range)	2.5 (0–10)	2.5 (0–7)	2 (1–5)	≤ 1	5.5 (0–10)	3.5 (1–6)	3.5 (2–5)		3 (1–6)
Living alone/with family member	16/31	4/6	3/4	2/4	4/4	2/7	–		1/6
Informal carers	Total	DE	IE^d^	IT	NL	NO	PT	SE	UK
Number of focus groups	21	5	3	2	2	2	2	2	3
Number of participants	96	21	16	8	10	7	11	8	15
Number of female participants	77	18	12	8	9	6	8	5	10
Mean age, years (range)	63 (37–91)	65 (47–83)	64 (50–89)	58 (49–70)	77 (68–86)	50 (37–66)	59 (43–78)	68 (56–91)	66 (46–84)
Mean caregiving time, years (range)	5 (1–12)	4.5 (1–10)	7 (2–12)	6 (2–10)	5.5 (1–11)	4 (1–9)	4 (0–8)	3 (2–7)	4 (1–10)
Relationship to person with dementia									
Spouse	47	10	9	–	10	2	4	2	10
Child	42	8	7	8	–	3	7	5	4
Other	7	3	–	–	–	2	–	1	1
Healthcare professionals	Total	DE	IE	IT	NL	NO	PT	SE	UK
Number of focus groups	20	4	3	2	2	2	2	2	3
Number of participants	114	16	18	9	12	13	12	12	22
Number of female participants	98	12	17	6	11	13	11	12	16
Mean age, years (range)	45 (23–62)	49 (42–60)	42 (28–56)	45 (26–52)	42 (27–63)	46 (25–61)	41 (27–56)	52 (44–62)	42 (23–57)
Mean working experience, years (range)	16 (0.25–42)	17 (3–30)	12 (2–25)	18 (1–26)	17 (3–35)	13 (0.25–30)	12 (2–30)	23 (7–42)	12 (0.25–30)
Professional backgrounds									
Nurses^a^	60	9	10	3	5	10	5	11	10
Social workers	6	3	1	1	–	–	1	–	–
General Practitioners	5	1	–	–	–	–	3	–	–
Other specialist physicians	6	1	2	–		–	–	–	4
Psychologists	10	–	–	1	6	–	1	–	2
Others^b^	27	2	5	4	1	3	2	1	6

^a^ Registered, assistant or community mental health nurses

^b^ Counsellors, educators, case managers

^c^ People with dementia living in institutional long-term care

^d^ *n* = 95 due to missing data

### Findings

Overall, we identified 16 categories describing influential aspects in accessing and using services that may serve as a barrier or facilitator. These categories were grouped into three global themes in order to structure the synthesis: 1) *Aspects related to the individuals involved* (characteristics of every individual involved), 2) *Aspects related to the health- and social care system* (such as available resources, regulations, features and design of services), and 3) *Overarching aspects* (important on both levels). Although the categories were derived separately for the three types of focus group, a considerable level of agreement, particularly in terms of the main categories, was revealed regarding barriers and facilitators to formal dementia care from the perspectives of people with dementia, informal carers and health and social care professionals (Table 2).

**Table 2:** Overview of (sub-) categories per type of focus group

Categories and subcategories			
	People with dementia	Informal carers	Health and Social Care Professionals
1) Aspects related to the individuals involved	1.1 Characteristics and situation of the informal carers		
	• *Attitude and beliefs towards formal care*	• *Being proactive* • *Mutual help between informal carers* • *Attitude and beliefs towards formal care and dementia* • *Personal motives, need factors & trigger situations*	• *Knowledge/information* • *Attitude and beliefs towards formal care and dementia* • *Emotions of the informal carer* • *Personal motives & crisis as a trigger* • *Financial resources*
	1.2 Characteristics and situation of the people with dementia		
	• *Attitude and beliefs towards formal care* ο *perceiving formal care as a threat to independence* • *Attitude and beliefs towards dementia/dealing with the disease* • *Being involved in decision making* • *Knowledge, information and experiences* • *Accepting the diagnosis & adaptation process* • *Being proactive/initiative of others* • *Personal motives to use formal care* • *Financial resources*	• *Attitude and beliefs towards formal care and dementia*	• *Attitude and beliefs towards formal care towards dementia* • *Emotions*
	1.3 Expectations of healthcare professionals and formal care		
	• *Lack of or uncertain expectations*	• *Lack of or uncertain expectations* • *Joint decisions and shared responsibilities* • *Sufficient information* • *Need-tailored support*	• *Firm diagnosis & reliable information* • *Emotional support* • *Understanding care needs*
	1.4 Experiences with the uptake of formal care		
	• *(Dis-)Satisfaction*	• *(Dis-)Satisfaction* • *Experiences towards the right moment of using formal care*	• *(Dis-)Satisfaction*
	1.5 Family structures and social environment		
	1.6 Characteristics & strategies of the health and social care professionals		
	• *Attitude towards dementia, people with dementia and towards informal carers* • *Relationship with health and social care professionals*	• *Attitude towards dementia, people with dementia and towards informal carers* • *Competencies (knowledge and social competences)* • *Trial visit*	• *Attitude towards dementia, people with dementia and towards informal carers* • *Competencies (knowledge and social competences)* • *Strategies of the health and social care professionals*
2) Aspects related to the (health and social care) system	2.1 Availability of services		
	• *Lack of services* • *Staff deficits and insufficient financing*	• *Lack of services* • *External barriers in utilisation (limited resources, insufficient financing, non-transparent structures)*	• *Lack of services (also regionally)* • *Staff deficits and insufficient financing* • *Time constraints*
	2.2 Features of the services		
	• *Need-tailored services* • *Key contact person*	• *Need-tailored services* • *Key contact person* • *Cost factor*	• *Need-tailored services*
	2.3 Complexity of the system		
	• *Complex regulations* • *Disjointed nature of the system*	• *Complex regulations* • *Disjointed nature of the system*	• *Complex regulations* • *Disjointed nature of the system* • *Variability of the system/unclear roles*
	2.4 Continuity		
	• *Key contact point/key contact person*	• *Key contact point/key contact person*	• *Key contact point/key contact person*
	2.5 Networking & collaboration		
	2.6 Role of the general practitioner (GP)		
3) Over-arching aspects	3.1 Information		
	3.2 Public awareness		
			3.3 Early contact

### Aspects related to the individuals involved

#### Characteristics and situation of the informal carer

The influence of the informal carers on formal care use was comprehensively discussed by the health and social care professionals and informal carers but was only seldom mentioned by the people with dementia. A *lack of knowledge and information* regarding dementia and available services may hinder people from seeking help. The informal carers’ *attitude and beliefs towards formal care* have a decisive impact and were predominantly described as reticent or negative, as seen from the perspectives of the health and social care professionals. Informal carers may not perceive a need for help and may not identify themselves as ‘carers’, particularly in the early stages of the disease.

*“It is, of course, a group that does not want to admit the problem, that there is something going wrong, and I see many cases where they try to conceal it as long as possible.”* (Healthcare professional/NL).

Some informal carers may feel obliged to care, perceive caregiving as a chance to give something in return to the person with dementia, or draw something positive out of caregiving.

*“…I feel a duty to take care of her (the person with dementia).”* (Informal carer/IT).

Thus, formal care may be experienced as interfering, and relinquishing care may be regarded as a personal failure. Expecting to be stigmatised due to dementia may further impede someone from seeking help. The informal carers may also be afraid of losing control and may find it difficult to accept strangers invading their privacy.

“*But they just have to get used to the idea that they (professional helpers) have to come in (the person’s house)*.” (Informal carer/DE).

Moreover, strong *emotions of the informal carer*, such as fear and anxiety (e.g., fear of being separated from their relative with dementia), may further contribute to non-utilisation. In contrast, service use is facilitated when informal carers are open-minded and *seek support proactively*. Moreover, *mutual help between informal carers* may enhance the utilisation of formal care through increasing motivation and sharing information. Informal carers may further have strong *personal motives* for using formal care, e.g., to remain in employment and to reduce burden.

*“The number of carers that approach us asking for support to assist their relative is increasing because they cannot miss work to provide care.”* (Healthcare professional/PT).

A crisis was regularly seen to be a *trigger for using formal care*, such as an accident, somatic disease, behavioural symptoms of the people with dementia, or the sudden inability of the informal carer to provide care.

*“Often, it does come to crisis. People can be plodding on quite nicely. One of them goes into hospital and then it’s a crisis. It would be much more useful being planned for.”* (Healthcare professional/UK).

Also, *the needs of the informal carers* and *the needs of the people with dementia* may trigger the use of formal care.

*“Unless there are others who see and hear what’s going on and share some of the responsibility, one is tense the whole time.” (Informal carer/SE).*

Depending on the healthcare system, the *financial resources* of the family may be a strong determinant. In IE, for example, private services were perceived to offer better-quality care for people with dementia, and thus, the ability to pay for private services may facilitate and accelerate access. In general, the financial circumstances of the families may influence the decision on whether formal care is used or not, particularly if private payment is required.

*“The problem is that the majority of us [carers] cannot afford private home care, and the services provided by the non-profit institutions do not meet our needs. For instance, support around the clock or during weekends is lacking.”* (Informal carer/PT).

#### Characteristics and situation of the person with dementia

*The attitude and beliefs of people with dementia towards the disease* and *towards formal care* also considerably influence the uptake of formal care. These attitudes were mainly seen as a great hindrance across all the countries, primarily from the perspectives of the health and social care professionals and the informal carers. People with dementia may have problems accepting the diagnosis, and because of the disease, they often lack awareness of their care needs.

*“We wish that she had much more help than she wants. But she doesn’t want to. That’s the case. Because she is not ‘ill’, and she is not ‘old’ either.*” (Informal carer/NO).

They further described that they felt a *lack of information* about their condition and about services available, particularly at the point of diagnosis. *Accepting the diagnosis and adaptation process* was described as being a long and demanding process, and seeking support during this emotionally burdensome phase may not be possible due to overwhelming *emotions*.

*“You’re diagnosed and thrown out into the big world. You don’t get told about any services.”* (Person with dementia/UK).

People with dementia clearly expressed their wish to remain independent and in control for as long as possible and, thus, to be *involved in decision-making* about their care. It emerged within these focus groups that formal care was considered a *threat to individual independence* by people with dementia and that it would only be accepted if it is perceived as absolutely necessary.

“*As a client, you must pay attention that you do not become too dependent […]*.” (Person with dementia/NL).

However, it also became apparent that people recognise that the moment will come when they have to rely on the *initiative of others* in finding help, and some participants reported retrospectively that the decision to use formal care had been prompted by others. Like the informal carers, people with dementia also have clear *personal motives to use formal care,* such as security issues or protecting family members from burdensome caregiving obligations or fulfilling social needs. This appeared to also be a strong motive for using services such as day care, where social activities take place, mutual support and social interaction can be experienced.

*“For me, it’s important that my children can carry on with their own lives.”* (Person with dementia/DE).

*“I always said I did not want to be a burden to my family. My children have so many concerns...work, their own kids.”* (Person with dementia/PT).

Some people with dementia in early stages or with early onset dementia reported that they tried to find help and support *proactively,* which facilitated access to services.

*“When I got the diagnosis, I contacted the home health care nurse at once because I knew that I would need help sooner or later. And I started with the pills because we started to be anxious […].”* (Person with dementia/NO).

#### Expectations of health and social care professionals and formal care

It became clear that people with dementia and informal carers had *no or only vague expectations* regarding formal care. Informal carers in particular describe more-general expectations, such as *sharing the responsibility* of caring for the person with dementia and help making *joint decisions* regarding the care*.* Thus, they expect *sufficient information* to be provided to them by health and social care professionals regarding the condition of the person with dementia, available services and financial support.

*“If you don’t know what you are looking for, it’s hard.”* (Informal carer/IE).

They expected that support would be *tailored to their needs,* including timely help, awareness of psychological and respite needs and the need for security of the person with dementia. Health and social care professionals were aware that knowing about and fulfilling these expectations may be helpful. They knew that *a firm diagnosis and reliable information* regarding the course of the disease, the legal aspects and the associated financial issues are expected of them. They further stated that people with dementia and their families may require *emotional support*, *understanding* of *their (care) needs* and respect for their personal situation.

#### Experiences with the uptake of formal care

The experiences with formal care may encourage continued use when *people are satisfied*, or these experiences may be off-putting and dissuade the families from using any further support when the contact with health and social care professionals was perceived as negative. The *right moment for using formal care* was controversial in the discussions. In retrospect, informal carers in some countries suggested that they started using professional help too late and that professional support should be used from the very beginning.

*“The right time is when a person receives the diagnosis, basically.”* (Informal carer/DE).

*“For Mum’s sake, give her a dignified life, not leave her isolated. As relatives, we should have requested help much earlier.”* (Informal carer/SE).

#### Family structures and social environment

The family structure and the social environment further influence the utilisation of formal care, which was revealed in all types of focus groups. If no one advocates for the person with dementia, it may take longer for the person to receive a diagnosis and the required support.

*“I think definitely accessing formal care is more often than not reliant on having somebody available, a carer, a friend, a neighbour, a child who is close by to encourage you to come forward.”* (Healthcare professional/UK).

Conversely, the existence of a social network that allows the distribution of care responsibilities can stabilise the caregiving situation at home and delay the uptake of services. A very close relationship between the person with dementia and the informal carer (particularly among couples) may be another barrier to using formal care.

*Healthcare professional A: “Exactly. ‘We’ve made a promise to each other’.”*

*Healthcare professional B: “‘We are going to be there for one another, no matter what happens. And now that’s what I’m sticking to’. You hear that relatively often, don’t you?”* (Healthcare professionals/DE).

*“Probably it’s me. I unconsciously […] feel the duty to take care of her. I mean, I feel the duty […]. I have always had a lot from my mother, being the only child.”* (Informal carer/IT).

Being an informal carer with further family obligations (e.g., caring for own children) triggers the use of formal care, while disputes among family members may either trigger or hamper the use of formal care, particularly those services typically decided upon by an informal carer.

#### Characteristics and strategies of the health and social care professionals

Health and social care professionals as individuals may serve as a barrier or facilitator, and how they behave plays a crucial role in the process of accessing and using formal care. If they express negative *attitudes and beliefs towards dementia or people with dementia* (e.g., that people with dementia are challenging care recipients or patronising behaviour) this may serve as a barrier, while respecting the person with dementia, considering their capacity and rights and addressing their needs, are considered facilitators.

*“I said to my own GP, I actually don’t want to see these doctors anymore because they are patronising. They are not listening to the information I’m giving them.”* (Person with dementia/IE).

“*And generally, anybody I would see is nearly in the later stages of dementia. Very few are in the early stages, where, you know, we could have had a lot more conversations of preventable work, and a lot of it is to do with the stigma and the GP not wanting to take that route.”* (Health care professional/IE).

Informal carers feel they need to be regarded as partners in caregiving, and their experience and knowledge should be valued.

*“I told the medical team that I was available to help with my father and explained to them that my presence soothed him, but they said it was not necessary.”* (Informal carer/PT).

Also of major importance are the *competencies of the health and social care professionals*, their *dementia-specific knowledge*, their *knowledge about the individual person* with dementia and their *social competencies*. Being sensitive to the needs of people, being open, supportive and empathic and creating an atmosphere of warmth and kindness were emphasised constantly across all focus groups. These aspects are particularly important in initial contact and for establishing a *good and trusting relationship*, which seemed to be a major facilitator.

*“[…] when the patient comes to our attention, it’s a strategic moment from all points of view […] So, this moment is a determining moment, even if the patient, or the relative, decides ‘I stay or I go away’.”* (Healthcare professional/IT).

*“It is so good to talk to the dementia nurse when I am sad. She makes me smile.”* (Person with dementia/NO).

Health and social care professionals apply a range of *strategies to enhance the use of formal care,* such as establishing a bond of trust. This can be done by, e.g., ensuring continuity in staff, keeping in regular and frequent contact, gradually increasing support, sharing decision making, constantly remaining approachable, and using reasonable arguments to persuade the informal carer, a strategy that was described as a sort of sales pitch by the health and social care professionals.

*“I always try to tell them [the relatives], ‘If you collapse tomorrow, you won’t be helping your relative whatsoever. He’d have to go into a nursing home straightaway. So you must ensure that you get some relief’.”* (Healthcare professional/DE).

### Aspects related to the health- and social care systems

#### Availability of services

*A lack of services* was revealed in all types of focus groups but was more deeply discussed by informal carers and health and social care professionals, particularly in the focus groups in DE, IE, NL, UK, PT and NO. Needed but lacking services were, for example, post-diagnostic support, night care, 24-h care on weekends, respite care at home or services for people with early onset dementia. Also, rigid criteria for accessing services create difficulties and limit the accessibility of services until the needs are significant.

*“The criteria for social services seem to be getting steeper and steeper. […] If they do an assessment, they’ll say well you don’t have sufficient needs for us to get involved.”* (Healthcare professional/UK).

*Insufficient financing* of services and *staff deficits* were described as major hindrances resulting in dissatisfaction. *Time constraints* of health and social care professionals are a result of these limited resources and were reported in the focus groups in most countries. Moreover, having enough resources and time was considered a facilitator.

*“And being able to have that time is also very positive for us as staff because then we are relaxed too and really take our time.”* (Healthcare professional/SE).

#### Features of the services

The accessibility and utilisation of services is determined by *needs-tailored support* that is flexible enough to meet the individual needs and by the *required payment*, i.e., services need to be affordable if they are not covered by insurance or are offered without out-of-pocket payment*.*

Most services are currently judged to be too disease-focused, and psychological and social needs are often not appropriately considered, particularly from the perspective of health and social care professionals and people with dementia. The co-location of dementia services with mental health services was reported to be potentially off-putting. However, meeting the needs of the person with dementia and the informal carer through formal care services can be quite challenging, as their needs may be divergent. For example, respite care can provide urgently needed relief for the informal carer but can be a burdensome experience for the person with dementia.

*“The experience for the person with dementia arriving in a respite setting can be very traumatising for them. They know that ‘I’m not in my own home, I’ve been brought here because Mary or John wants to go on holidays or they just need a break’ […].”* (Healthcare professional/IE).

From the perspectives of people with dementia and informal carers, having a single *key contact person* was an important characteristic of services or service providers in easing access.

#### Complexity of the system

The complexity of the system was reported as a considerable hindrance to access to formal care. Examples were *complex regulations* and bureaucracy; *disjointed nature of the system*; too many care options with poorly defined responsibilities; system inconsistency and service inequity across the country, causing a high degree of *variability and unclear roles* among professionals; or simply involving too many different health and social care professionals in supplying care.

*“… those safe key boxes. Somebody recently said that they had given the number to 13 people. Would you give your number and the key access to your house to 13 people?”* (Healthcare professional/IE).

Although health and social care professionals acknowledged that a wide range of diverse services is needed to meet the needs of the person with dementia and their families, the complexity of services and regulations has to be reduced or people need better guidance in order to facilitate access to services.

#### Continuity

Avoiding fragmented services and unnecessary breaks (e.g., between the private and public sectors, acute and long-term care or health and social care), ensuring that the same staff take care of the person with dementia and establishing ongoing contact with people with dementia and their families—not just during a crisis—would contribute to continuity and facilitate access to formal care. Having a ‘*key contact point/key contact person*’ was described as an important aspect of continuity and was mentioned by all stakeholders across the countries. While such a person is clearly reported to be lacking in most countries, the dementia coordinators in NO, which are in place in many municipalities across the country, seem to fulfil this function.

*“The best thing I know is when we come in so early that it is not necessary to bring in anyone else yet. When one actually can make a difference […] in relation to preparing for what’s to come.”* (Healthcare professional/NO).

*“Well, I think a key worker role. I think a key worker role and a point of contact. So in other words, every time I give a diagnosis in the clinic, I should be able to say, ‘Your point of contact is X.*” (Health care professional/IE).

#### Networking and collaboration

Ensuring good collaboration among health and social care professionals emerged in the focus groups in all countries as an important facilitator that was deemed to contribute to appropriate and timely resource allocation.

*“The network should simply work better. Often, the first way is to the neurologist or physician. But this has to be better coordinated.*” (Informal carer/DE).

*“It is so good here because we all work so close to one another, so you can go to the care administrator and ask, Is this person on the register here? If not, it may be relevant soon.”* (Healthcare professional/Sweden).

#### The role of the general practitioner (GP)

The GP is usually considered the first contact point. The GP makes a diagnosis or refers the patient to specialists, provides information and assumes an initial gate-keeping function. However, GPs do not always fulfil their role in providing access to services in an appropriate way or consistently across the country. For example, they may not have sufficient knowledge about dementia or might be inattentive regarding first symptoms.

*“I have to say, when I went to him* [the GP] *and I said, ‘I’m a bit worried about [husband’s] memory,’ do you know what he said to me? ‘Yes, the last time he was with me I thought there was something strange.’ Now the last time could have been six months before that. But he didn’t think it was worth his while to pick up the phone and say, ‘Do you think there’s anything funny going on with your husband?”* (Informal carer/IE).

### Overarching aspects

#### Information

A need for better information emerged in most of the focus groups. Participants called for sufficient, clear, precise and comprehensible information about the disease, the available services, and legal issues. This information is a crucial facilitator of access to services.

*“First, they* [informal carers] *don’t know, “Where should I go? Who should I ring?” They know that it exists, but how does one do it* [apply for formal care]*?”* (Healthcare professional/SE).

Information should be easily accessible and, ideally, delivered by a key contact person who can select appropriate information and repeat as necessary.

*“They have to have known what was going on. But nobody ever approached us.”* (Informal carer/IE).

#### Public awareness

Improved public awareness, for example, supported by appropriate media campaigns, may contribute to the normalisation and de-stigmatisation of people with dementia. It may also prevent banalisation of the disease and thus enhance the use of formal care.

*“Everyone talks about AIDS, tuberculosis and other diseases, but when it comes to Alzheimer’s, they say, Oh, the forgetfulness, it is part of the ageing process”.* (Informal Carer/PT).

#### Early contact

Early contact was considered an important facilitator from the perspectives of health and social care professionals and as a precondition to provide need-driven care instead of sudden crisis intervention. Early contact provides time to establish a bond of trust, to understand the family’s capacity to care and to get to know the person with dementia and their family as individuals.

*“As early as possible, simply so that one can think about which system should be set up, what the relatives can realistically cope with. As early as possible.”* (Healthcare professional/DE).

“[…] *They have to have known what was going on. But nobody ever approached us.*” (Informal carer/IE).

While the view prevailed that contact should be established as early as possible, the timely use of formal care was considered quite dependent on individual circumstances.

*“The right moment to start care is a combination of the estimated risk of danger and especially: what is the quality of life of this person with dementia?”* (Healthcare professional/NL).

## Discussion

To the best of our knowledge, this is the first study to combine the perspectives of all the main stakeholders involved in the process of accessing and using formal care in dementia. While the majority of previous qualitative research has focused solely on the perceptions of informal carers, our study adds the perspectives of people with dementia. Accepting the diagnosis and adaptation process was described as being a long and demanding process, and seeking support during this emotionally burdensome phase may not be possible, due to overwhelming emotions. People with dementia expressed their wish to remain independent and in control for as long as possible and, thus, to be involved in decision-making related to their care. Former research has revealed that formal care can be an ambiguous gain. While it can include positive aspects, such as relief of the informal carer, it may also increase uncertainty, affect self-perception or disturb the relationship with the care recipient [31]. This also seems to be true for people with dementia. On the one hand, they describe certain benefits of formal care, such as creating security or protecting the family from caregiving obligations, but on the other hand, they perceive formal care as a threat to their independence. This perceived threat to independence was described as a major barrier in using formal care, since it conflicts with the desire to stay independent and to protect autonomy, i.e., factors that are closely related to the quality of life of people with dementia [32]. Thus, they often tried to avoid formal care as long as possible. Moreover, the fact that the perspectives of people with dementia were only rarely discussed by healthcare professionals and informal carers, who mainly referred to the reluctance of people with dementia and their lack of insight into the disease, may show that people with dementia are still not equally included in the decision making process. Although participation and living independently are accepted principles in treating people with dementia—principles that have been promoted by advocacy organisations for many years [33, 34]—the transfer to everyday practice is still challenging, as our findings indicate. Insufficient consideration of these principles still occurs across Europe and may hamper access to and the use of needed and beneficial formal care services.

The major influence of the attitudes and beliefs of the people with dementia and informal carers emerged as a principal aspect, one that was revealed in all three types of focus group. Accordingly, the decision to use formal care may not be based merely on objective or perceived needs but may also be influenced by a complex interplay of psychological and social aspects [6, 35–37]. An adaptation of the widely applied Behavioral Model of Health Service Use [38] aims to explain the use of long-term care services, and it supports the influence of psychosocial factors. The adapted model is based of qualitative focus groups with African-Americans and Caucasians and suggests that psychosocial aspects play a central role in explaining long-term service use, which may mediate the influences cultural backgrounds [36]. A recent meta-synthesis shows that comparable hindrances are experienced across people with culturally and linguistically-diverse backgrounds, such as lack of knowledge or stigma related to mental health, but theses aspects may be exaggerated by cultural habits [39]. We did not attempt to investigate and compare cultural differences across the participating European countries. However, we revealed tendencies that may reflect differences between countries. For example, family caregiving obligations and stigma around mental health were more clearly revealed within the focus groups in the Southern countries PT and IT than in the North of Europe, especially in NO.

To successfully overcome these major barriers, our study suggests that early and constant contact with a health or social care professional is essential—irrespective of the country. Such a key contact person should proactively approach the families as early as possible. This person needs to create a trusting relationship with the people with dementia and their families; to recognise individual needs, beliefs and attitudes; to provide information; and to offer counselling. Moreover, this person should be approachable to the families throughout the process of the disease. Interestingly, research shows that informal carers of people with dementia retrospectively regret not having used services earlier [40], which was also confirmed in our study. Informal carers and people with dementia often have a lack knowledge about services, are unaware of the benefits thereof or do not consider themselves as informal carers or as being in need of formal support. A key contact person who better knows the individual families could appropriately address these aspects and better coordinate the range of different services that may be required when dementia progresses. The Actifcare study shows that only in Norway was such a key contact person (in terms of a dementia care team implemented in most of the municipalities) regularly available [22]. This is important, as many countries have implemented case management approaches that may be beneficial for people with dementia and their informal carers [41] but that are not necessarily equal to the key contact person. Moreover, case management approaches do not automatically enhance the desire to stay independent of the person with dementia, which seems to be a key aspect to enhance access to formal care for people with dementia. Advance care planning may be a possible strategy to better engage people with dementia and enhance their autonomy with regard to using formal care [42]. However, advance care planning has so far predominantly been applied in institutional long-term care, mainly with a focus on end-of-life care, and evidence is lacking for people with dementia living within the community [42].

Beyond that, the findings of our European study confirm research findings regarding access to and use of formal care, such as a lack of knowledge regarding available services, misconceptions and stigma related to dementia, lack of services that focus on social needs and a complex healthcare systems that is difficult to navigate, all of which have been reported over the last decade [6, 9, 13, 43, 44]. Our study shows that these barriers still occur in practice and that there is an urgent need to develop and implement appropriate strategies and mechanisms to overcome these barriers. This is even more important when considering that the majority of European countries have already launched national dementia strategies in order to appropriately support people with dementia and their informal carers [45].

### Strengths and limitations

Unlike many studies, our qualitative study was not limited to informal carers but also included a considerable number of people with dementia across eight European countries. The latter group has been widely neglected so far in qualitative research addressing access to and utilisation of formal dementia care.

The consistency and methodological rigor of the analysis process across the countries were ensured by developing a manual that included a clear description of the data collection and analysis procedures. Furthermore, we aimed to provide a detailed and comprehensive report of the methodological steps. The strategy was shown to be practical and feasible and may contribute to a methodological discussion on how to deal with qualitative data in transnational research. All steps were developed in close collaboration with all partners, and the synthesis of the national reports was counterchecked by the national research teams in order to avoid misinterpretations, deviations or omissions. Overall, quite comparable categories describing barriers and facilitators were found across the types of groups and across the countries, supporting the credibility of the analysis.

Our study also has limitations that need to be acknowledged. A predefined number of two focus groups per type were included, which is slightly below the recommended number. This decision had to be made due to the overall schedule of the European project. Some of the (sub-) categories were not reported in all national analyses, and it can only be speculated whether these aspects are unimportant in these countries or whether they were not revealed due the limited number of focus groups.

Moreover, the cross-national synthesis necessarily had to be broader and could not explore in depth certain country-specific, individual or service-related aspects that may have an important impact.

Furthermore, the majority of healthcare professionals had a nursing background, reflecting the large workforce of nurses in various sectors and functions within the long-term dementia care system [46, 47]. Social care professionals and GPs were underrepresented. This may be a limitation because GPs in particular are an important first contact point when accessing formal dementia care [22], and they are the preferred source of help, following close family members [11]. The Actifcare project focused on formal long-term services for people with dementia and their families and did not investigate the voluntary sector [19]. Correspondingly, the focus groups were organised with paid health care professionals, who may have neglected an important perspective.

According to the sampling criteria, people who had had any kind of experience with accessing or using formal care were included. Thus, we left out the group of people who never attempted to access formal support. A considerable number of younger people with dementia were included. Their perspectives may be overrepresented by focusing predominantly on expectations prior to using formal care. This group also has considerably different needs, for example, a changing family structure with teenage children [48], and their method of access and their experiences in using formal services may differ from those of older people with dementia. In SE, no focus group with people with dementia could be conducted. In PT, people with dementia living in a long-term care institution were included because formal community support for people with dementia is widely lacking, and institutional long-term care often constitutes the first experience with formal care. Nevertheless, these participants were able to provide valuable insights from a Portuguese perspective.

The definition of formal care used within the Actifcare study was quite broad. Thus, the findings provide a general overview of barriers and facilitators to the access to and use of formal care. Focusing on specific types of formal care and phases of dementia would offer a deeper and more focused understanding of barriers and facilitators that are important in specific situations.

Finally, our findings have to be interpreted in light of the current political and economic situation of the eight countries. Within the Dutch focus groups, for example, a considerable level of uncertainty was expressed by the participants due to the forthcoming legal changes and expected financial cuts [49]. The economic austerity faced in IE and PT may have increased the negative expectations of available services [50, 51], while the current legal changes in DE are likely to improve the situation of people with dementia and their families [52].

## Conclusion

Based on our findings, a health and social care professional who serves as a key contact person could address major barriers in the access to and use of formal care for people with dementia and their informal carers. Contact with people with dementia and their families should be initiated proactively and as early as possible, and a trusting and consistent relationship needs to be established. Further investigations are needed on how the concept of a key contact person can be integrated with existing case management approaches and how the independence and autonomy of people with dementia can be strengthened when formal care needs to be accessed and used. This may be a key facilitator regarding enhanced access to formal care for people with dementia and their families.

## Additional file

Additional file 1: Actifcare_topic guide. The topic guide which was used in the focus groups across all countries. (DOCX 47 kb)

## Abbreviations

Actifcare: ACcess to TImely Formal Care
DE: Germany
GP: General Practitioner
IE: Ireland
IT: Italy
NL: The Netherlands
NO: Norway
PT: Portugal
SE: Sweden
UK: United Kingdom
