# Supplementary material for: Barriers and facilitators to the access to and use of formal dementia care: findings of a focus group study with people with dementia, informal carers and health and social care professionals in eight European countries
Source: BMC Geriatr. 2018 Jun 4;18:131. doi: 10.1186/s12877-018-0816-1 (PMC5987478; doi:10.1186/s12877-018-0816-1)
Supplement: Supplementary file 1 — Actifcare_topic guide. The topic guide which was used in the focus groups across all countries. (DOCX 47 kb) [file 12877_2018_816_MOESM1_ESM.docx]

**
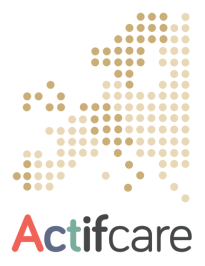
WP2 Focus groups: Topic guide**

| **Steps** | **Questions for the person with dementia** | **Questions for the carer** | **Questions for the healthcare professional** |
| --- | --- | --- | --- |
| **Opening** | *Please introduce yourself (interviewer can give an example).*  *Please tell us who supports you at home and which services you are using.* | *Please introduce yourself with your name, and describe in a few sentences how the care is organized in your case: Who supports you apart from family and friends?* | *Please introduce yourself with your name and professional background.* |
| **Introduction** | *In which situation have you realised that further support would be good, in addition to family and friends?* | *Please remember the time when you first thought about professional care and support. Please tell us about this time.* | *Please describe in a few sentences your work in the praxis: What do you do so that people with dementia and their carers get professional support?* |
| **Transition** | *How was it for you when you realised that you need support in addition to family and friends?* | *What did you do to get support? Please describe how you have found professional support.*  *Probing (only to be asked in case the topic is not discussed spontaneously):*   - *Where or who did you turn to?* - *How did you find this service/contact person?* - *Who supported you?* - *How was that for you?* | *Please think about how you get into contact with people with dementia and their carers.*  *[Please ask separate questions]*  *What are the challenges?*  *What makes it easier?*  *Please describe a situation in which people with dementia and their carers did not use any professional support, albeit you considered it necessary.* |
| **Key questions** | *What makes it easier to get support other than from your family and friends?*  *What makes getting support difficult other from your family?* | *At the very beginning: How were your thoughts and feelings regarding professional care and support?*    *Probing (only to be asked in case the topic is not discussed spontaneously):*  *What were your expectations regarding professional support?* | *What do people with dementia and their carers expect from you when they get in contact, looking for professional support?* |
|  | *How is for you to get support outside your family?*  *What is most important for you?* | *Please describe how it was when you got professional support for the first time. What was especially important during that phase?* | *Please think about the phase when people with dementia and their carers use professional support at home for the first time (e.g.: home care services or day care centre). What is especially important during this phase?* |
|  | *For you, when is the right moment for using support from outside the family?* | *For you, when is the right moment for using professional support from outside the family?* | *Against the background of your experience: When is the right moment for using professional support?* |
| **Final/Closing questions** | *Which type of support outside your family is most important for you?* | *What would help you in practice (or what would have helped you) to find suitable professional support?* | *What would help you in practice to facilitate the access to professional support for people with dementia and their carers?* |
|  | *Is there anything else you would like to tell us? Something we did not speak about?* | *Did we forget anything? Is there something we should have talked about but didn’t?* | *Did we forget anything? Is there something we should have talked about but didn’t?* |
|  | *May be asked after the first focus groups in order to improve the process and the organisation.*  *Many thanks for sharing your experiences with us. Now we have a last request. This was the first in a series of interviews. Is there anything we could improve or anything we should change?* | *May be asked after the first focus groups in order to improve the process and the organisation.*  *Many thanks for sharing your experiences with us. Now we have a last request. This was the first in a series of interviews. Is there anything we could improve or anything we should change?* | *May be asked after the first focus groups in order to improve the process and the organisation.*  *Many thanks for sharing your experiences with us. Now we have a last request. This was the first in a series of interviews. Is there anything we could improve or anything we should change?* |
